# Postbiotics as Antiinflammatory and Immune‐Modulating Bioactive Compounds in Metabolic Dysfunction‐Associated Steatotic Liver Disease

**DOI:** 10.1002/mnfr.202400754

**Published:** 2024-11-05

**Authors:** Yusuf Yilmaz

**Affiliations:** ^1^ Department of Gastroenterology School of Medicine Recep Tayyip Erdoğan University Rize Türkiye; ^2^ The Global NASH Council Washington DC 53020 USA

**Keywords:** gut microbiota, immunomodulation, inflammation, metabolic dysfunction‐associated steatotic liver disease, nutritional interventions, postbiotics

## Abstract

Postbiotics, defined as products or metabolic byproducts secreted by live bacteria or released after bacterial lysis, are emerging as promising therapeutic agents for metabolic dysfunction‐associated steatotic liver disease (MASLD). This review explores the antiinflammatory and immunomodulatory properties of various postbiotics, including exopolysaccharides, lipoteichoic acid, short‐chain fatty acids, hydrogen sulfide, polyamines, tryptophan derivatives, and polyphenol metabolites. These compounds have demonstrated potential in mitigating steatotic liver infiltration, reducing inflammation, and slowing fibrosis progression in preclinical studies. Notably, postbiotics exert their beneficial effects by modulating gut microbiota composition, enhancing intestinal barrier function, optimizing lipid metabolism, reducing hepatic inflammation and steatosis, and exhibiting hepatoprotective properties. However, translating these findings into clinical practice requires well‐designed trials to validate efficacy and safety, standardize production and characterization, and explore personalized approaches and synergistic effects with other therapeutic modalities. Despite challenges, the unique biological properties of postbiotics, such as enhanced safety compared to probiotics, make them attractive candidates for developing novel nutritional interventions targeting the multifactorial pathogenesis of MASLD. Further research is needed to establish their clinical utility and potential to improve liver and systemic outcomes in this increasingly prevalent condition.

## Graphical abstract

Postbiotics, derived from bacterial products and byproducts, show promise in treating metabolic dysfunction‐associated steatotic liver disease. They offer benefits like antiinflammatory and immunomodulatory effects, improve lipid metabolism, reduce hepatic inflammation and steatosis, enhance gut microbiota composition, strengthen intestinal barriers, and decrease oxidative stress. These properties enable postbiotics to effectively target the complex pathogenesis of this liver condition.

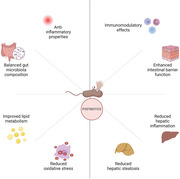

## 1 Introduction

The concept of postbiotics is emerging as a significant area of interest within the functional food sector, underscoring the role of gut microbiota in health through the secretion of bacterial components and functional microbial metabolites.^[^ ^1^, ^2^ ^]^ According to the International Scientific Association for Probiotics and Prebiotics (ISAPP), postbiotics refer to *preparations of inanimate microorganisms and/or their components that confers a health benefit on the host*.^[^ ^3^ ^]^ However, this definition has sparked debate,^[^ ^4^ ^]^ with alternative perspectives suggesting that *soluble factors (products or metabolic byproducts), secreted by live bacteria, or released after bacterial lysis*, can be considered postbiotics.^[^ ^5^ ^]^ Within this expanded framework, postbiotics are understood as functionally active, bacterial‐derived compounds that do not necessarily require the presence of inactivated microbial biomass. Although beneficial effects are not a mandatory criterion within this definition,^[^ ^5^ ^]^ numerous postbiotics have demonstrated considerable potential for promoting health across diverse nutritional contexts.^[^ ^6^ ^]^

From a mechanistic standpoint, postbiotics contribute to host‐microbiome balance by maintaining eubiosis and preserving the integrity of the intestinal mucosal barrier.^[^ ^7^, ^8^ ^]^ Emerging evidence also suggests that certain postbiotics may attenuate inflammatory signaling and modulate immune responses,^[^ ^9^ ^]^ thereby alleviating chronic low‐grade inflammation,^[^ ^10^, ^11^ ^]^ a critical factor in the pathogenesis of non‐communicable diseases.^[^ ^12^ ^]^ Additionally, postbiotics have been shown to positively interact with the host's innate and acquired immune responses, potentially enhancing pathogen response and maintaining homeostasis.^[^ ^9^ ^]^ Collectively, these properties can positively influence host metabolic pathways via the gut–liver axis,^[^ ^13^ ^]^ positioning postbiotics as promising candidates for the prevention and treatment of metabolic dysfunction‐associated steatotic liver disease (MASLD), which affects 38.2% of the global population and is a leading cause of liver‐related morbidity and mortality.^[^ ^14^ ^]^

MASLD is a complex, multifactorial disorder characterized by hepatic steatosis in conjunction with at least one cardiometabolic risk factor, in the absence of other discernible causes of steatotic liver infiltration.^[^ ^15^ ^]^ Conversely, steatosis accompanied by inflammation and hepatocyte injury is classified as metabolic‐associated steatohepatitis (MASH).^[^ ^15^ ^]^ The increasing prevalence of obesity and metabolic syndrome has contributed to the substantial health burden posed by MASLD, necessitating innovative strategies to expand current therapeutic options.^[^ ^16^ ^]^ Postbiotics, with their diverse biological actions,^[^ ^1^, ^2^ ^]^ demonstrate considerable potential in targeting key pathophysiological underpinnings of MASLD/MASH.^[^ ^17^ ^]^ Notably, postbiotics offer several advantages over living probiotics, including longer shelf life, enhanced safety profiles, and no risk of infection for frail or immunocompromised individuals.^[^ ^1^, ^2^, ^9^, ^13^ ^]^ Moreover, they can be administered alongside antibiotics without concerns about transmitting resistance genes.^[^ ^18^ ^]^

Building on these premises, this review aims to explore the principal postbiotics in relation to their antiinflammatory and immune‐modulating properties and how they can be harnessed to counteract the pathogenic factors associated with MASLD. Understanding the role of postbiotics in this context may facilitate the development of novel nutritional interventions to combat this increasingly prevalent hepatic condition through the food–microbiota axis.

## 2 Experimental Section

### 2.1 Search Strategy

This narrative review sought to provide a critical overview of the antiinflammatory and immunomodulatory properties of postbiotics and their potential application in combating MASLD. References were sourced from searches conducted on PubMed for peer‐reviewed articles published in English between January 1, 2004, and August 1, 2024. The search terms included “postbiotics” AND “inflammation”, “immune system”, “liver”, “steatosis”, “steatohepatitis”, “MASLD”, “MASH”, and “non‐alcoholic fatty liver disease”. Additionally, the bibliographies of relevant papers were examined. Only articles published in English were considered for inclusion.

### 2.2 Classification of Postbiotics

**Figure 1:**
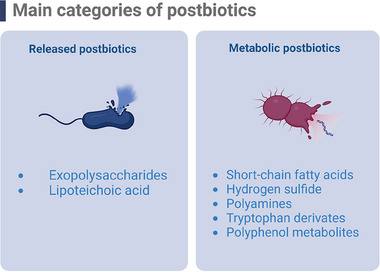
Classification of postbiotics. Left panel: Released postbiotics include exopolysaccharides and lipoteichoic acid. Right panel: Metabolic postbiotics encompass short‐chain fatty acids, hydrogen sulfide, polyamines, tryptophan derivates, and polyphenol metabolites (Created in BioRender.com).

In this study, we adopted a broad definition of postbiotics as proposed by Aguilar‐Toalá et al.^[^ ^5^ ^]^ For clarity in presentation, postbiotics were categorized based on their origin into two distinct groups,^[^ ^19^ ^]^ as follows: 1) released postbiotics, such as exopolysaccharides (EPSs) and lipoteichoic acid (LTA), and 2) metabolic postbiotics, which are soluble factors produced through microbial metabolic pathways in the intestine (**Figure** **1**). The discussion of each type of postbiotic will focus on their potential to target MASLD, emphasizing their biological activities (**Table** **1**). Additionally, the potential applications of complex postbiotic mixtures will be explored (**Table** **2**).

**Table 1:** Summary of different postbiotic types, their sources or origins, and their effects on metabolic dysfunction‐associated steatotic liver disease.

Postbiotic type	Source/origin	Effects on MASLD
Exopolysaccharides	Synthesized and released by bacteria	Enhance intestinal barrier function, reducing bacterial translocation to the liver, preventing inflammatory responses and metabolic dysfunction associated with MASLD
Lipoteichoic acid	Found in Gram‐positive bacteria, extracted from inactivated probiotics	Reduces TLR4 signaling, benefiting lipid metabolism and reducing intestinal permeability, which helps manage MASLD by mitigating bacterial translocation and inflammation
Short‐chain fatty acids	Produced during fermentation of inulin and fructooligosaccharides by gut microbiota	Improve insulin sensitivity and promote fatty acid oxidation. SCFAs, like butyrate, activate AMPK in hepatocytes, reducing lipid accumulation and potentially alleviating hepatic steatosis and steatohepatitis in MASLD
Hydrogen sulfide	Produced by sulfate‐reducing bacteria in the gut, including *Desulfovibrio vulgaris*	Impaired H_2_S production is linked to MASLD pathogenesis. H_2_S donors can suppress inflammation and oxidative stress, alleviating MASLD symptoms
Polyamines	Synthesized by intestinal bacteria like *Bacteroides* spp. and *Fusobacterium* spp.	Spermidine reduces hepatic lipid accumulation, insulin resistance, and inflammation. It also activates AMPK and autophagy, beneficial in managing MASLD
Tryptophan derivatives	Metabolized by gut microbiota into indole compounds	Indole‐3‐acetic acid and indole‐3‐propionic acid improve insulin resistance, lipid metabolism, and reduce liver steatosis and inflammation, alleviating MASLD
Polyphenol metabolites	Transformed by gut bacteria from dietary polyphenols	Urolithins and equol maintain intestinal barrier, counteract gut dysbiosis, and suppress lipid metabolic reprogramming, beneficial in managing MASLD by reducing inflammation and supporting liver metabolism

AMPK, AMP‐activated protein kinase; H_2_S, hydrogen sulfide; MASLD, metabolic dysfunction‐associated steatotic liver disease; SCFA, short‐chain fatty acid; TLR4, toll‐like receptor 4.

**Table 2:** Animal studies investigating the effects of postbiotic mixtures, derived from various probiotic strains, on liver‐related disorders.

Study	Postbiotic source	Model	Key findings	Therapeutic potential
Pan et al.^[^ ^21^ ^]^	*Lacticaseibacillus paracasei* CCFM1224	Mice	Curbed weight gain, enhanced lipid metabolism, reduced hepatic steatosis and inflammation, increased the abundance of *Akkermansia muciniphila*	Protective against MASLD
Song et al.^[^ ^23^ ^]^	Oat‐based postbiotics with *Lactiplantibacillus plantarum*	Rats	Alleviated liver injury, improved fatty acid metabolism, reduced inflammation, enhanced fatty acid profiles	Nutritional therapeutics for metabolic dysfunctions
Ye et al.^[^ ^91^ ^]^	*Lactiplantibacillus plantarum*‐derived postbiotics	Mice	Increased survival rate of alcohol‐treated hepatocytes, restored serum liver enzymes and lipid levels, increased the abundance of *Akkermansia muciniphila*	Protective against acute alcoholic liver injury
Cheng et al.^[^ ^92^ ^]^	*Limosilactobacillus reuteri*	Mice	Modulated bile acid metabolism, regulated FXR pathway, mitigated ethanol‐induced liver damage	Protective against in alcohol‐induced hepatic steatosis

FXR, farnesoid X receptor; MASLD, metabolic associated steatotic liver disease.

## 3 Released Postbiotics

### 3.1 Exopolysaccharides

EPSs are high‐molecular‐weight polymeric compounds, which can be either soluble or insoluble.^[^ ^24^ ^]^ These extracellular substances play a vital role in safeguarding the producing organisms from desiccation, phagocytosis, and osmotic stress.^[^ ^25^ ^]^ Chemically, EPSs are composed of repeated sugar moieties and are often substituted with proteins, lipids, and noncarbohydrate groups – including phosphate, succinate, acetate, glycerol, pyruvate, and sulfate.^[^ ^26^ ^]^ Recently, EPSs produced by probiotic lactic acid bacteria have been extensively investigated for their health‐promoting activities, particularly in light of their “generally recognized as safe” regulatory status.^[^ ^27^ ^]^ In this context, Xu et al.^[^ ^28^ ^]^ examined whether EPSs from *Lentilactobacillus buchneri* TCP016 could reduce d‐galactosamine‐induced liver damage in mice by modulating gut microbiota. The EPSs, comprising rhamnose, xylose, glucosamine, glucuronic acid, galactose, galacturonic acid, glucose, and mannose in specific molar ratios, have been shown to significantly reduce hepatic enzyme levels and proinflammatory cytokines. Notably, these postbiotic molecules improved intestinal mucosal integrity and strengthened the intestinal barrier, thereby reducing bacterial translocation to the liver.^[^ ^28^ ^]^ This property of EPSs from lactic acid bacteria is particularly relevant in the context of MASLD, where increased intestinal permeability leads to the translocation of detrimental molecules such as lipopolysaccharides (LPSs) from Gram‐negative bacteria, triggering inflammatory responses and metabolic dysfunction in the liver.^[^ ^29^ ^]^ In this context, research by Zhou et al.^[^ ^30^ ^]^ demonstrated that EPS from *Lactiplantibacillus plantarum* NCU116 can enhance intestinal barrier function by upregulating tight junction proteins like zonulin and occludin. Interestingly, a decreased expression of these proteins in intestinal epithelial cells is closely linked to the development and progression of steatotic liver disease.^[^ ^31^ ^]^ Collectively, these findings indicate that EPSs possess considerable potential as postbiotics with hepatoprotective properties, achieved through the modulation of inflammatory responses and the enhancement of intestinal barrier function.

### 3.2 Lipoteichoic Acid

LTA is a surface‐associated amphiphilic molecule found in Gram‐positive bacteria, known for its role in bacterial adhesion.^[^ ^32^ ^]^ As a postbiotic, LTA can be extracted from inactivated probiotic microorganisms using methods such as sonication, heating, enzymatic processes, or chemical treatments.^[^ ^33^ ^]^ The immunomodulatory properties of LTA vary significantly depending on the bacterial strain from which it is derived. For instance, LTAs from *Limosilactobacillus fermentum* and *Lacticaseibacillus casei* can induce proinflammatory responses by increasing the expression of tumor necrosis factor‐alpha (TNF‐α).^[^ ^34^ ^]^ In contrast, LTAs from *L. plantarum* A3, *Limosilactobacillus reuteri* DSMZ 8533, and *Lactobacillus acidophilus* CICC 6074 have been shown to reduce proinflammatory cytokine secretion while enhancing the production of the antiinflammatory cytokine IL‐10.^[^ ^35^ ^]^ Notably, LTA derived from *L. reuteri* DSMZ 8533 has been shown to inhibit the expression of the mitogen‐activated protein kinase (MAPK) and nuclear factor kappa‐B (NF‐κB) pathways,^[^ ^35^ ^]^ which are crucial for the inflammatory response triggered by LPS and are also implicated in the pathogenesis of MASLD.^[^ ^36^, ^37^ ^]^ Recent evidence also suggests that LTA may suppress toll‐like receptor 4 (TLR4)‐mediated signaling.^[^ ^38^ ^]^ This is particularly relevant for its potential application in managing MASLD, as hepatic TLR4 expression is commonly upregulated in this condition due to increased fatty acid levels and LPS exposure.^[^ ^39^ ^]^ Inhibiting TLR4 can enhance fatty acid oxidation in the liver,^[^ ^40^ ^]^ indicating that LTA's ability to downregulate TLR4 signaling might benefit lipid metabolism. Importantly, LTA possesses the ability to enhance mucin expression, thereby reducing intestinal permeability.^[^ ^41^ ^]^ This property could be especially advantageous in MASLD, given the significant role of bacterial translocation and the gut–liver axis in the disease's pathogenesis.^[^ ^31^ ^]^

## 4 Metabolic Postbiotics

### 4.1 Short‐Chain Fatty Acids

Short‐chain fatty acids (SCFAs) – including acetate, propionate, and butyrate – are crucial postbiotics produced during the fermentation of inulin and fructooligosaccharides by gut microbiota in the colon.^[^ ^42^ ^]^ While Bacteroidetes primarily generate acetic acid and propionate, Firmicutes predominantly produce butyrate.^[^ ^43^ ^]^ Once produced, SCFAs are absorbed by colonocytes and enter systemic circulation, where they exert significant metabolic effects, including potent antiinflammatory actions.^[^ ^44^ ^]^ Butyrate, for example, inhibits the NF‐κB signaling pathway, leading to a reduction in proinflammatory cytokines such as TNF‐α, IL‐6, and IL‐1β in macrophages and peripheral blood mononuclear cells.^[^ ^45^, ^46^ ^]^ Additionally, SCFAs modulate both innate and adaptive immunity by inhibiting histone deacetylases in immune cells, thereby influencing cytokine production and chemotaxis.^[^ ^47^ ^]^ In addition, they play a crucial role in regulating T cell differentiation, promoting the development of antiinflammatory regulatory T cells and Th17 cells over proinflammatory Th1 and Th2 responses.^[^ ^47^ ^]^ Notably, butyrate may enhance gut immunity by promoting antimicrobial peptide production by Paneth cells.^[^ ^48^ ^]^ Emerging research suggests that SCFAs may hold therapeutic potential for MASLD. Deng et al.^[^ ^49^ ^]^ reported that SCFA supplementation reduces hepatic steatosis and steatohepatitis in mice fed a methionine‐ and choline‐deficient diet. Mechanistically, butyrate activates AMP‐activated protein kinase (AMPK) in hepatocytes, promoting fatty acid oxidation and reducing lipid accumulation.^[^ ^49^ ^]^ In addition, SCFAs may improve insulin sensitivity,^[^ ^50^ ^]^ which is typically diminished in MASLD.^[^ ^14^ ^]^ Recently, Thing et al.^[^ ^51^ ^]^ observed elevated levels of SCFAs in a cohort of 100 patients with MASLD. However, this phenomenon is likely compensatory, as acetate and propionate contribute to maintaining low‐grade inflammation through their effects on circulating immune cells.^[^ ^44^ ^]^ Correspondingly, another study reported a decrease in fecal SCFA levels in MASLD patients with significant fibrosis, while no significant difference was observed in those with moderate MASLD.^[^ ^52^ ^]^ Interestingly, Yoon et al.^[^ ^53^ ^]^ reported that the probiotics *Bifidobacterium breve* and *Bifidobacterium longum* attenuate MASLD in animal models primarily through the production of SCFAs as postbiotics. This finding should encourage further human studies to explore the role of probiotic‐derived SCFAs as a potential complementary strategy for managing MASLD in clinical settings.

### 4.2 Hydrogen Sulfide

Sulfate‐reducing bacteria in the human gut are capable of producing hydrogen sulfide (H_2_S),^[^ ^54^ ^]^ which functions as a gaseous signaling molecule within the body.^[^ ^55^ ^]^ Due to its solubility in lipophilic solvents, H_2_S can readily penetrate cell membranes.^[^ ^56^ ^]^ This postbiotic may serve as an electron acceptor in anaerobic bacterial respiration and acts as an inhibitor of butyrate oxidation.^[^ ^57^ ^]^ The role of H_2_S in inflammation remains controversial, with varying results likely due to differences in the use of endogenous versus pharmacological hydrogen sulfide across diverse animal models and cell culture systems.^[^ ^58^ ^]^ However, the role of H_2_S in the immune system appears to be more consistently documented. A reduction in H_2_S has been associated with the development of spontaneous autoimmune diseases or the acceleration and worsening of various immune‐mediated diseases.^[^ ^59^ ^]^ In addition, therapeutic delivery of low amounts of H_2_S via small molecule donors may enhance the function of various immune cells and protect them against dysfunction induced by various noxious stimuli.^[^ ^60^ ^]^ Interestingly, there is growing evidence that impaired H_2_S production is involved in the pathogenesis of MASLD. Studies have shown that hepatic H_2_S biosynthesis is impaired in methionine‐ and choline‐deficient diet‐induced rat models of MASLD^[^ ^61^ ^]^ and that hepatic H_2_S levels are significantly lower in high‐fat diet (HDF)‐fed mice compared to control animals.^[^ ^62^ ^]^ Additional animal experiments have demonstrated that treatment with H_2_S prevents hepatic steatosis by suppressing inflammation and reducing oxidative stress.^[^ ^63^ ^]^ Similarly, Wu et al.^[^ ^64^ ^]^ reported that the administration of H_2_S alleviated experimental MASLD by inhibiting apoptosis and promoting autophagy. Gut bacteria responsible for H_2_S production include mucus‐associated species of bacterial genera belonging to the Desulfovibrionaceae and Enterobacteriaceae families.^[^ ^65^ ^]^ Notably, Hong et al.^[^ ^66^ ^]^ revealed that *Desulfovibrio vulgaris*, a potent generator of H_2_S,^[^ ^65^ ^]^ was effective in attenuating hepatic steatosis in HFD‐fed mice, demonstrating significant anti‐MASLD effects. However, it is important to note that an overgrowth of H_2_S‐producing bacteria may be pathogenic,^[^ ^65^ ^]^ suggesting that further research is necessary to fully understand the risks and benefits of this gaseous postbiotic.

### 4.3 Polyamines

Polyamines (PAs) are aliphatic polycations that are essential in regulating gene expression, cell proliferation, and differentiation.^[^ ^67^ ^]^ The most prevalent PAs produced by intestinal bacteria, acting as postbiotics, are putrescine, spermidine, and spermine.^[^ ^68^ ^]^ In the human gut, *Fusobacterium* spp. and *Bacteroides* spp. are capable of synthesizing spermidine and putrescine, resulting in concentrations between 0.5 and 1 mM in the intestinal lumen of healthy subjects.^[^ ^68^ ^]^ Notably, the synthesis of PAs is contingent upon the availability of amino acid precursors or other intermediates.^[^ ^69^ ^]^ Furthermore, the bacterial uptake of PAs is regulated by highly conserved, membrane transport systems.^[^ ^70^ ^]^ In the context of MASLD, spermidine is the most extensively studied PA. Beyond its well‐documented antioxidant properties, spermidine has been shown to mitigate the LPS‐induced production of nitric oxide and downregulate cyclooxygenase‐2 expression.^[^ ^71^ ^]^ Additionally, spermidine imparts antiinflammatory properties to macrophages by inducing mitochondrial superoxide‐dependent AMPK activation and autophagy.^[^ ^72^ ^]^ A study by Szydlowska et al.^[^ ^73^ ^]^ revealed a decrease in PA levels during the progression of MASH in both human and murine feces. Moreover, spermidine was observed to normalize the number of Kupffer cells in the livers of mice with NASH; however, these beneficial effects did not translate into improvements in liver steatosis or fibrosis severity.^[^ ^73^ ^]^ In a separate study, Ni et al.^[^ ^74^ ^]^ reported that spermidine supplementation significantly reduced insulin resistance, steatosis, steatohepatitis, and fibrosis in a murine models of MASH induced by a Western diet. This was achieved through autophagy activation and thyroid hormone‐responsive protein signaling.^[^ ^74^ ^]^ Furthermore, research by Zhang et al.^[^ ^75^ ^]^ suggested that spermidine mitigates MASLD by suppressing ferroptosis, as evidenced by decreased levels of iron and reactive oxygen species. Finally, Zhou et al.^[^ ^76^ ^]^ demonstrated that spermidine partially restores protein synthesis and mitochondrial function in experimental MASH, and prevents MASH progression in vivo through enhanced hepatic protein synthesis.

### 4.4 Tryptophan Derivates

Approximately 5% of tryptophan introduced with the diet is metabolized by the gut microbiota into various derivatives, including indole compounds.^[^ ^77^ ^]^ These postbiotics are garnering attention for their antiinflammatory and immunomodulatory properties,^[^ ^78^ ^]^ offering potential therapeutic benefits for MASLD. Among these metabolites, indole‐3‐acetic acid (I3A) has been shown to mitigate hepatotoxicity induced by an HFD in mice, improving lipid metabolism, insulin resistance, and reducing oxidative and inflammatory stress.^[^ ^79^ ^]^ I3A also alleviates diet‐induced metabolic impairments, such as hepatic steatosis and glucose dysmetabolism, by correcting mitochondrial respiration defects.^[^ ^80^ ^]^ In obese humans, I3A levels increase postbariatric surgery and are negatively correlated with liver fat attenuation.^[^ ^81^ ^]^ Notably, Ding et al.^[^ ^82^ ^]^ demonstrated that oral administration of I3A significantly reduced hepatic steatosis and inflammation in a mouse model of MASLD, decreased hepatic triglycerides and serum ALT, and modulated the liver metabolome and proteome by reducing enzymes involved in hepatic lipogenesis. Mechanistically, I3A's antiinflammatory effects are mediated through AMPK activation in macrophages.^[^ ^82^ ^]^ Another tryptophan metabolite, indole‐3‐propionic acid (IPA), is implicated in MASLD pathogenesis. Sehgal et al.^[^ ^83^ ^]^ found that lower circulating IPA levels were associated with liver fibrosis and inflammation in obese individuals, particularly those without type 2 diabetes, and correlated with genes involved in hepatic stellate cell (HSC) activation and fibrosis signaling. In vitro experiments also showed that IPA reduced HSC adhesion, migration, and activation, suggesting its therapeutic potential in liver fibrosis management.^[^ ^83^ ^]^ In a separate study, Zhao et al.^[^ ^84^ ^]^ demonstrated that IPA administration in rats fed an HFD maintains intestinal epithelium homeostasis, reduces inflammation by inhibiting NF‐κB signaling, and lowers proinflammatory cytokines. Additionally, IPA supplementation improves glucose metabolism, enhances insulin sensitivity, inhibits liver lipid synthesis and inflammation, and maintains intestinal homeostasis,^[^ ^85^ ^]^ thereby potentially alleviating MASLD.

### 4.5 Polyphenol Metabolites

Only 5%–10% of ingested polyphenols are absorbed in the upper gastrointestinal tract, while 90%–95% reach the colon, where they are transformed by resident bacteria into bioavailable metabolites.^[^ ^86^ ^]^ Among these polyphenol metabolites, urolithins^[^ ^87^ ^]^ and equol^[^ ^88^ ^]^ are notable postbiotic compounds with potential therapeutic applications in MASLD. Accordingly, Xu et al.^[^ ^89^ ^]^ have shown that urolithin C, a gut metabolite derived from ellagic acid‐containing foods, demonstrates protective effects against MASLD in mice fed a choline‐deficient amino acid‐defined HFD by maintaining intestinal mucosal barrier and counteracting gut dysbiosis. Similarly, Zhang et al.^[^ ^20^ ^]^ reported that urolithin A exerts antisteatotic effects in a fructose‐induced MASLD mouse model by suppressing lipid metabolic reprogramming and triggering lipophagy through the AMPK pathway. In addition to their direct effects on liver metabolism, urolithins have demonstrated antiinflammatory properties by suppressing the NF‐κB signaling pathway in various cell types.^[^ ^87^ ^]^ Equol, a phytoestrogenic postbiotic synthesized by gut bacteria from the soy isoflavone daidzein, has shown antiinflammatory activity by suppressing inflammatory responses, reducing the expression IL‐6, and inhibiting LPS‐induced TLR4 activation.^[^ ^88^ ^]^ Interestingly, men with lean NAFLD have been shown to have low rates of equol production.^[^ ^90^ ^]^ In addition, the gut microbiota composition differed significantly between equol producers and nonproducers, suggesting a potential link between equol production, gut microbiota, and lean NAFLD pathogenesis.^[^ ^90^ ^]^

## 5 Postbiotic Mixtures

In the emerging landscape of innovative therapies for MASLD, postbiotic mixtures are gaining recognition as a promising approach, offering multifaceted therapeutic potential that includes modulating gut microbiota, regulating lipid metabolism, and exerting antiinflammatory and immunomodulatory properties. Pan et al.^[^ ^21^ ^]^ explored the effects of postbiotics derived from *Lacticaseibacillus paracasei* CCFM1224 on MASLD in mice. The findings revealed that the tested postbiotic mixture, when given alongside an HDF, effectively curbed weight gain, enhanced lipid metabolism, and reduced hepatic steatosis and inflammation.^[^ ^21^ ^]^ Notably, it increased the relative abundance of *Akkermansia muciniphila*,^[^ ^21^ ^]^ a mucin‐degrading bacterium known for its protective effects against metabolic disorders.^[^ ^22^ ^]^ Additionally, postbiotics influenced liver metabolic pathways and gene expression related to lipid metabolism, suggesting its potential as a protective agent against MASLD.^[^ ^21^ ^]^ Song et al.^[^ ^23^ ^]^ developed innovative oat‐based postbiotics fermented with *L. plantarum* and other probiotics to investigate their effects on rats with long‐term high‐sucrose consumption. The results revealed that these postbiotics alleviated liver injury, improved fatty acid metabolism, reduced inflammation, and enhanced fatty acid profiles in the liver, highlighting their potential as nutritional therapeutics for metabolic dysfunctions.^[^ ^23^ ^]^ Notably, preclinical evidence indicates that postbiotic mixtures could be potentially beneficial for individuals with MetALD. In the current nomenclature framework, this category refers to those with MASLD who consume alcohol in quantities exceeding 140 g per week for women and 210 g per week for men.^[^ ^15^ ^]^ Accordingly, Ye et al.^[^ ^91^ ^]^ investigated the protective effects of *L. plantarum*‐derived postbiotics on acute alcoholic liver injury. The authors found that preincubation with postbiotics significantly increased the survival rate of alcohol‐treated HL7702 human hepatocytes and, in vivo, presupplementation with postbiotics‐loaded calcium alginate hydrogel restored serum liver enzymes and lipid levels in mice after acute alcohol intake. Additionally, postbiotics presupplementation upregulated genes involved in fatty acid metabolism, and enhanced the abundance of *A. muciniphila*.^[^ ^91^ ^]^ Recently, Cheng et al.^[^ ^92^ ^]^ conducted a study examining the effects of postbiotics derived from *L. reuteri* on alcohol‐induced hepatic steatosis in mice, with a focus on elucidating their mechanisms of action. The research identified the farnesoid X receptor (FXR) as a critical target for these postbiotics, which modulate bile acid metabolism by affecting enterohepatic circulation. The authors concluded that postbiotics significantly mitigated ethanol‐induced liver damage by regulating the FXR pathway.^[^ ^92^ ^]^

## 6 Discussion

**Figure 2:**
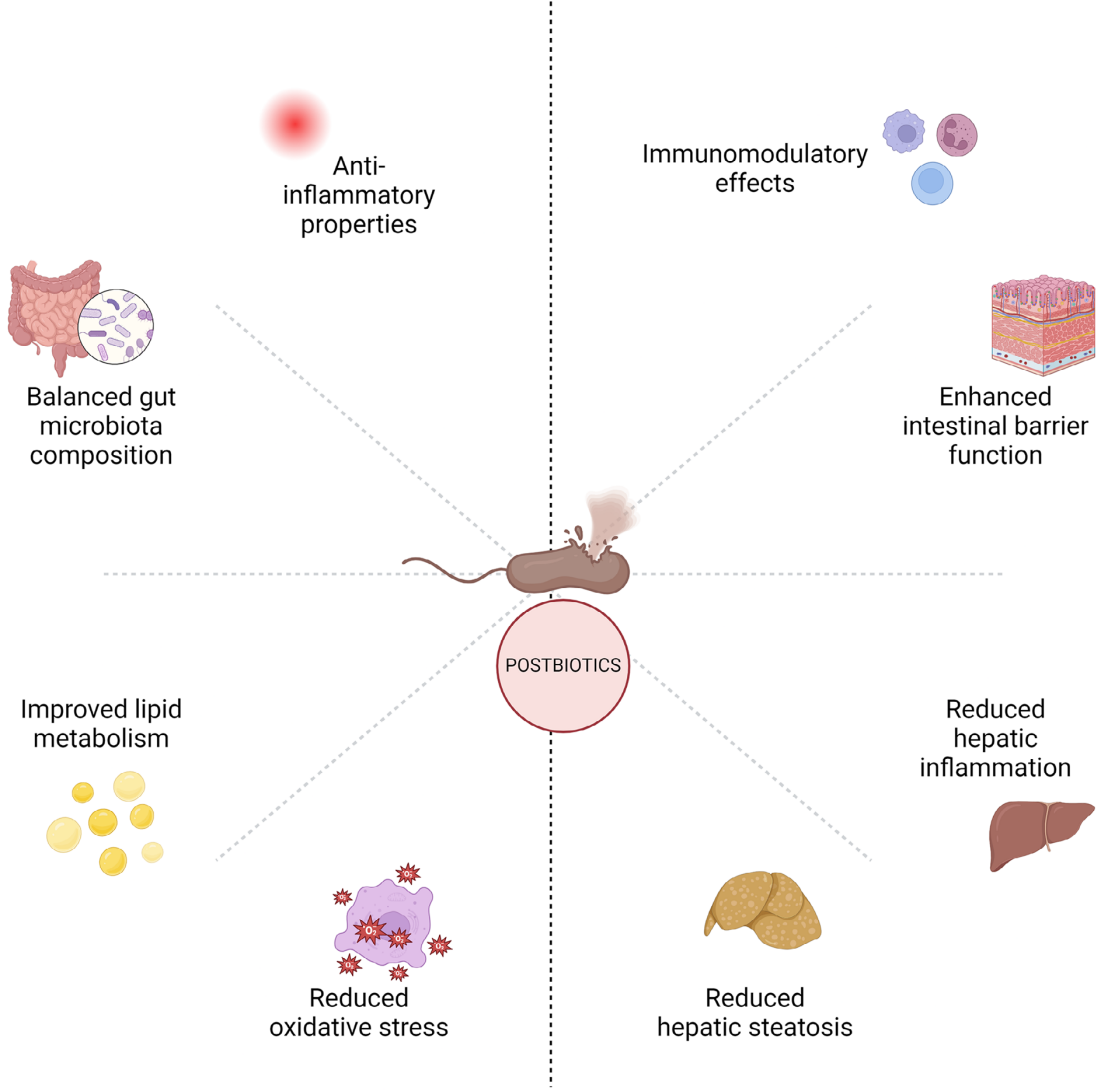
Schematic representation of the diverse biological activities of postbiotics. This diagram illustrates the wide‐ranging effects of postbiotics on various aspects of metabolic and hepatic health. The central node represents postbiotics, with radiating connections to eight key biological activities (Created in BioRender.com).

The escalating global burden of MASLD, coupled with the limited availability of FDA‐approved treatments and difficulties in sustaining lifestyle changes, highlights the pressing need for innovative management strategies.^[^ ^93^ ^]^ In this context, postbiotics, due to their diverse biological activities (**Figure** **2**), emerge as promising candidates for addressing the key pathogenic mechanisms underlying this increasingly prevalent chronic liver condition. The potential benefits and challenges associated with postbiotic interventions will now be critically examined.

### 6.1 Harnessing Postbiotics Against MASLD Histological Hallmarks

MASLD is histologically characterized by hepatic steatosis.^[^ ^15^ ^]^ Its inflammatory subtype, MASH, is further distinguished by the presence of lobular and portal inflammation.^[^ ^15^ ^]^ Over time, these inflammatory changes can progress to fibrosis, potentially leading to cirrhosis and other severe liver complications.^[^ ^17^ ^]^ Significantly, all three key histological hallmarks of MASLD can be targeted by compounds within the postbiotics category. In the context of steatotic liver disease, butyrate emerges as a promising candidate due to its ability to negatively regulate hepatic lipogenesis, primarily through the activation of the AMPK pathway.^[^ ^94^ ^]^ Conversely, H_2_S can decrease lipid accumulation within the liver parenchyma by inducing hepatic lipolysis.^[^ ^95^ ^]^ Spermidine has the potential to reduce steatosis through autophagy activation and thyroid hormone‐responsive protein signaling.^[^ ^74^ ^]^ Additionally, I3A^[^ ^82^ ^]^ and urolithin A^[^ ^20^ ^]^ can decrease hepatic triglyceride content, suppress lipid metabolic reprogramming, and trigger lipophagy. Regarding hepatic inflammation in MASH, LTA from *L. reuteri* DSMZ 8533 may inhibit the proinflammatory NF‐κB signaling pathway,^[^ ^35^ ^]^ a property shared with both IPA,^[^ ^84^ ^]^ a tryptophan derivative, and polyphenol metabolites, such as urolithins.^[^ ^87^ ^]^ In terms of fibrosis, a critical determinant of adverse patient outcomes,^[^ ^96^ ^]^ spermidine stands out as a promising antifibrotic postbiotic. It activates HSCs and inhibits the deposition of extracellular matrix proteins through autophagy activation.^[^ ^97^ ^]^ Similar effects on HSCs can also be elicited by IPA.^[^ ^83^ ^]^

### 6.2 MASLD Heterogeneity and Postbiotics: Potential Tools for Personalized Medicine

Patients with MASLD demonstrate considerable variability in disease trajectories, marked by both histological diversity and varied clinical outcomes.^[^ ^98^ ^]^ Although some individuals may remain at the benign steatotic liver disease stage, others may advance to severe conditions such as fibrosis, cirrhosis, and hepatocellular carcinoma.^[^ ^98^ ^]^ This heterogeneity is partly due to significant interindividual differences in gut microbiota, which can impact the integrity of the intestinal epithelial barrier and facilitate the translocation of proinflammatory molecules to the hepatic parenchyma via the gut–liver axis.^[^ ^99^ ^]^ In a precision medicine framework, where management and treatment are tailored to the unique characteristics of each patient,^[^ ^100^ ^]^ postbiotic formulations can be customized based on specific imbalances in an individual's gut microbiota composition. For example, patients with a deficiency in SCFA‐producing bacteria might benefit from SCFAs as postbiotics to restore microbial balance. Conversely, soy isoflavones could be used to enhance equol production in patients whose gut microbiota contains bacteria capable of metabolizing these compounds.^[^ ^88^ ^]^ The type and dosage of postbiotics can also be adjusted according to the severity of MASLD. For instance, individuals with significant fibrosis and low circulating IPA levels^[^ ^83^ ^]^ may be ideal candidates for treatment with this tryptophan derivative. This personalized approach ensures that “precision postbiotics”^[^ ^101^ ^]^ are optimized to meet the specific needs of each MASLD patient, potentially reducing the risk of fibrotic changes and improving clinical outcomes.

### 6.3 Postbiotics and Functional Food in MASLD

The promising effects of postbiotics in MASLD are further supported by the benefits observed with certain functional foods, such as kefir and kombucha, which naturally contain postbiotics as a result of their fermentation processes.^[^ ^102^ ^]^ Kefir, a fermented milk drink produced using kefir grains, is notable for its content of a specific postbiotic EPS termed kefiran.^[^ ^103^ ^]^ Kefiran has potent immunomodulatory effects, restoring the balance between T helper 1 and T helper 2 cells,^[^ ^103^ ^]^ an action that may be particularly beneficial in the context of MASLD.^[^ ^104^ ^]^ Additionally, kefir consumption in experimental models has been shown to reduce hepatic steatosis by inhibiting the expression of several genes involved in lipogenesis – including sterol regulatory element‐binding protein 1, fatty acid synthase, and acetyl‐CoA carboxylase.^[^ ^105^ ^]^ Kombucha, a low‐alcoholic beverage derived from plant materials (such as tea, juices, and herb extracts) and a symbiotic culture of acetic acid bacteria, lactic acid bacteria, and yeasts, develops a rich postbiotic composition during fermentation, including SCFAs.^[^ ^106^ ^]^ Similar to kefir, kombucha has been repeatedly shown to alleviate MASLD in several rodent models.^[^ ^107^, ^108^ ^]^ Collectively, these findings suggest that both kefir and kombucha could be valuable functional foods in the management of MASLD, offering potential therapeutic benefits through their postbiotic content.

### 6.4 Safety of Postbiotics

Despite encouraging preclinical findings, the translation of postbiotics from bench to bedside for patients with MASLD necessitates thorough safety testing, as outlined by the ISAPP guidelines.^[^ ^3^ ^]^ These recommendations emphasize the need for a detailed characterization of postbiotic preparations and a comprehensive evaluation of safety for the intended use in the target host.^[^ ^3^ ^]^ Moreover, it is crucial that postbiotics are produced and stored under conditions that ensure their stability and purity.^[^ ^3^ ^]^ It is also noteworthy that, despite the myriad potential health benefits and detoxifying effects of postbiotics,^[^ ^109^, ^110^ ^]^ certain types may pose specific safety concerns. For example, high levels of PAs can be toxic, as their catabolism can lead to the production of reactive aldehydes and hydrogen peroxide, which can damage proteins, DNA, and other cellular components.^[^ ^111^ ^]^ Therefore, it will be essential to carefully control both the dosage and duration of PA delivery to avoid potential toxicity. Similarly, the use of H_2_S as a postbiotic must be approached with caution. Its concentration should be thoroughly regulated to prevent toxicity, as chronic exposure to H_2_S has been associated with adverse effects on respiratory, ocular, and neurological health.^[^ ^112^ ^]^ By adhering to the ISAPP safety guidelines,^[^ ^3^ ^]^ researchers and manufacturers can effectively harness the potential benefits of postbiotics against MASLD while minimizing associated risks.

### 6.5 Limitations and Future Directions

The evidence gathered from preclinical models provides promising support for the potential utility of postbiotics in treating steatotic liver disease. However, it is important to acknowledge that existing animal models do not fully capture the complexities of human MASLD.^[^ ^113^ ^]^ Despite this limitation, which necessitates caution in interpreting preclinical findings, animal models, when combined with in vitro experiments, remain indispensable tools for informing future clinical research, offering invaluable mechanistic insights. Beyond the previously outlined safety considerations, several other issues must be addressed before postbiotics can be routinely used in clinical practice. Standardizing postbiotic formulations presents certain difficulties, including variability in composition and concentration across different strains and batches, limited understanding of specific bioactive components and their mechanisms of action, potential instability during processing and storage, and the lack of validated bioanalytical methods.^[^ ^114^ ^]^ To address these challenges, several strategies should be implemented, including 1) establishing clear definitions and classification frameworks for postbiotics; 2) developing standardized production processes and analytical methods to ensure consistency; 3) identifying and purifying specific bioactive components; 4) conducting comprehensive stability testing; and 5) establishing validated analytical assays and clinically relevant biomarkers.^[^ ^114^, ^115^ ^]^ Another area that warrants attention is the delivery of postbiotics.^[^ ^116^ ^]^ Innovative oral formulations and nanoparticle‐based delivery systems represent promising strategies to enhance the in vivo administration of postbiotics for the treatment of MASLD. Oral delivery methods, such as pH‐sensitive systems utilizing enteric coatings, colon‐targeted delivery, and mucoadhesive formulations, can safeguard postbiotics from degradation in the gastrointestinal tract, facilitate targeted release in the intestines, and improve absorption.^[^ ^116^ ^]^ Nanoparticle carriers, including lipid nanoparticles, polymeric nanoparticles, and mesoporous silica nanoparticles, can encapsulate postbiotics to enhance their stability, bioavailability, and liver‐specific targeting.^[^ ^116^ ^]^ Following these considerations, well‐designed clinical trials will be essential to confirm the efficacy and safety of postbiotics in diverse patient populations with MASLD. These studies should determine optimal dosing regimens and evaluate clinical and patient‐reported outcomes. Furthermore, investigating the potential synergistic effects of postbiotics with other management strategies – including prebiotics, probiotics, lifestyle modifications, dietary interventions, and pharmacological agents – will be crucial to fully explore their potential in managing MASLD.

## 7 Conclusion

Postbiotics represent a promising frontier in the development of novel nutritional interventions for MASLD. By leveraging their antiinflammatory and immunomodulatory properties, postbiotics have the potential to target the multifactorial pathogenesis of this increasingly prevalent hepatic condition. However, further clinical research is essential to establish their safety and efficacy in human trials and to optimize strategies for clinical application. This could ultimately lead to improved clinical and patient‐reported outcomes, providing a valuable addition to the therapeutic arsenal against steatotic liver disease.

## Conflict of Interest

The author declares that the research was conducted in the absence of any commercial or financial relationships that could be construed as a potential conflict of interest.

## Data Availability

Data sharing is not applicable to this article as no new data were created or analyzed in this study.
